# A Meta-Transcriptomics Survey Reveals Changes in the Microbiota of the Chinese Mitten Crab *Eriocheir sinensis* Infected with Hepatopancreatic Necrosis Disease

**DOI:** 10.3389/fmicb.2017.00732

**Published:** 2017-04-26

**Authors:** Huaishun Shen, Yanan Zang, Kun Song, Yuanchao Ma, Tianhao Dai, Ali Serwadda

**Affiliations:** ^1^Key Laboratory of Freshwater Fisheries and Germplasm Resources Utilization, Ministry of Agriculture, Freshwater Fisheries Research Center, Chinese Academy of Fishery SciencesWuxi, China; ^2^Wuxi Fisheries College, Nanjing Agricultural UniversityNanjing, China

**Keywords:** *Eriocheir sinensis*, hepatopancreatic necrosis disease, tenericutes, bacteroidetes, meta-transcriptomics

## Abstract

Infection of the freshwater Chinese mitten crab *Eriocheir sinensis* with hepatopancreatic necrosis disease (HPND) has been a major problem in the crab-cultivated Chinese Province of Jiangsu since 2015. To explore the etiology of HPND, meta-transcriptomic libraries of the hepatopancreata from crabs with and without HPND were constructed. Comparison analyses showed that there were no statistically significant differences in viral and microsporidial communities in the hepatopancreata of diseased and healthy crabs. Bacteroidetes, Proteobacteria, and Firmicutes were the most dominant bacterial phyla in the hepatopancreata of healthy crabs, with a combined prevalence of 93%. However, a decrease in bacterial diversity and a striking shift in the microbial composition were found in the hepatopancreata of crabs infected with HPND. Tenericutes was the most prevalent bacterial phylum in diseased crabs (31.82%), whereas its prevalence was low in healthy crabs (0.02%). By contrast, the prevalence of Bacteroidetes was significantly lower in crabs with HPND (3.49%) than in crabs without HPND (41.04%). We also found that the prevalence of Actinobacteria was higher in crabs with HPND (16.70%) than in crabs without the disease (4.03%). The major bacterial family within the Tenericutes phylum in crabs with HPND was detected by polymerase chain reaction and determined to be Mycoplasmataceae. In conclusion, there were striking changes in the microbiota of diseased and healthy crabs. Specifically, the prevalence of bacteria belonging to Tenericutes and Actinobacteria phyla increased, whereas the prevalence of bacteria belonging to the Bacteroidetes phylum decreased in crabs with HPND, clearly pointing to an association with HPND.

## Introduction

The freshwater Chinese mitten crab *E. sinensis* is an economically important crustacean cultured in the Chinese provinces of Jiangsu, Anhui, Hubei, and Liaoning (Shen et al., 2014). With the rapid increase of the *E. sinensis* aquacultural industry, numerous diseases have recently evolved, thus resulting in huge economic losses (Shen et al., 2015). For example, hepatopancreatic necrosis disease (HPND, “shuibiezi” in Chinese), which affects *E. sinensis*, has been a major problem in the crab-cultivated area of Jiangsu Province since 2015. Crabs with HPND exhibit multiple clinical symptoms, including hepatopancreata that are lighter in color (turning from golden yellow and light yellow to gray–white) and soft shells that are darker in color than usual, as well as muscle atrophy and edema (Ding et al., 2016). Furthermore, the stomachs and intestines are empty in many diseased crabs. The majority of the diseased crabs do not die immediately; instead, they continue to molt, albeit at a later stage of breeding. However, these crabs are of little/no economic value, and they eventually die. A previous study reported that microsporidia were detected in the hepatopancreata of crabs infected with HPND (Ding et al., 2016). In addition, hepatopancreatic injury caused by environmental toxicants is believed to be one of the main causes of HPND. However, the etiology of HPND is unknown.

The hepatopancreas of crustaceans functions in both digestion and absorption (Wang et al., 2004b). A dense microbial colonization has been observed in the hepatopancreas of several isopods (Wood and Griffiths, 1988; Zimmer et al., 2001; Wang et al., 2004b) and the shrimp *Neocaridina denticulate* (Cheung et al., 2015). In addition to nutrition and digestion, the immanent microbiota play crucial roles in the immune response of their animal hosts (Cheung et al., 2015), and an imbalance of host–microbiota homeostasis has been shown to be responsible for certain illnesses (Chen et al., 2015). A previous study has reported symbiotic bacteria in crabs to belong to the phyla Bacteroidetes, Proteobacteria, Firmicutes, and Tenericutes (Li et al., 2007; Givens et al., 2013; Chen et al., 2015; Zhang et al., 2016). Of these symbiotic bacteria, Tenericutes are unique, because they lack a cell wall, are small in size, and possess a reduced genome (Ryan and Ray, 2004). Furthermore, bacteria of the genus *Candidatus Hepatoplasma*, which belong to the Tenericutes phylum, were identified in symbionts colonizing the midgut glands of terrestrial isopods, and a positive correlation was identified between the survival of hosts and the ingestion of low-quality food (Wang et al., 2004a, 2007; Fraune and Zimmer, 2008; Leclercq et al., 2014). In addition, bacteria of the phylum Tenericutes have been shown to associate with several crustacean diseases, such as mycoplasma spp., which was isolated from the moribund prawn *Peneaux monodon* (Ghadersohi and Owens, 1999); spiroplasma, which was linked to tremor disease in the Chinese mitten crab (Wang et al., 2003); and *Acholeplasma* sp., which was implicated in clearwater disease of the mud crab *Scylla serrata* (Chen et al., 2011).

In this study, meta-transcriptomic libraries were constructed from the hepatopancreata of crabs with and without HPND, and viral, microsporidial, and bacterial communities were compared between the two groups. We report herein on statistically significant changes in the microbiota of diseased and healthy crabs. Our data provide valuable insights on the etiology of HPND.

## Materials and methods

### Sample collection

In June 2015, two crabs with clinical symptoms typical of HPND and two healthy crabs were obtained from an aquatic breeding pond in Yandu District, Yancheng City, Jiangsu Province, China for meta-transcriptomic sequencing. In June 2016, more than 100 crabs exhibiting clinical symptoms typical of HPND were obtained from several aquatic breeding ponds in Jiangdu District, Yangzhou City and Yandu District, Yancheng City, Jiangsu Province, China.

### cDNA library construction and illumina sequencing

Total RNA from the hepatopancreata of two crabs infected with HPND, as well as two healthy crabs, was isolated using the RNeasy® Plus Mini Kit (Qiagen, Valencia, CA, USA) according to the manufacturer's protocol. To remove genomic DNA, RNA was treated with RNase-free DNase (Qiagen, Germany) according to the manufacturer's protocol. The quality and quantity of the total RNA was estimated with a NanoDrop 2000 Spectrophotometer (Thermo Scientific, USA) and an Agilent 2100 Bioanalyzer (Agilent Technologies, Palo Alto, CA, USA). RNA integrity was assessed by electrophoresis on a 1% agarose gel. In order to ensure that there was no contamination of the genomic DNA, 1 μg RNA was used as PCR template, the expression of the β-actin gene of *Eriocheir sinensis* (accession number: HM053699.1) was selected as a control, using the primer pair *EsACTIN*-F (GCATCCACGAGACCACTTACA) and *EsACTIN*-R (CTCCTGCTTGCTGATCCACATC). The PCR program was as follows: denaturation at 94°C for 3 min; 35 cycles of 94°C for 30 s, 58°C for 30 s, and 72°C for 1 min; and 72°C for 10 min. Ribosomal RNA (rRNA) was depleted using the Ribo-Zero™ Magnetic Kit (Epicenter, Charlotte, NC, USA). The cDNA library was prepared using the TruSeq™ RNA Sample Prep Kit (Illumina, San Diego, CA, USA). The quality of the cDNA library was assessed using an Agilent 2100 Bioanalyzer. Sequencing was carried out on a HiSeq 2500 Ultra-High-Throughput Sequencer using the Mid Output Kit (both from Illumina), and 150-bp pair-end reads were obtained for each run.

### Meta-transcriptomic data analysis

The analysis of the entire meta-transcriptome was carried out as follows: the quality of each raw read (Phred score) was evaluated using the FastQC toolkit (http://www.bioinformatics.babraham.ac.uk/projects/fastqc/), and the adaptor was eliminated with SeqPrep software (https://github.com/jstjohn/SeqPrep). Thereafter, low-quality bases (Phred score < 20) were trimmed, and reads shorter than 50 bp were discarded with Sickle software (https://github.com/najoshi/sickle). rRNA reads were discarded after alignment to SILVA SSU (16S/18S) and SILVA LSU (23S/28S) databases with SortMeRNA software (http://bioinfo.lifl.fr/RNA/sortmerna/). The resulting high-quality reads were then used in the subsequent assembly. The mega-transcriptome was *de novo* assembled with Trinity software (http://trinityrnaseq.github.io/; version trinityrnaseq-r2013-02-25) using default parameters as previously described (Grabherr et al., 2011). ORFs were predicted using TransGeneScan software (http://sourceforge.net/projects/transgenescan/). Non-redundant gene catalogs were constructed with an identity of 95% and a coverage of 90% using CD-HIT software (http://www.bioinformatics.org/cd-hit/). To evaluate the expression level of each transcript, the FPKM (fragments per kilobase of exon per million fragments mapped) value of each transcript was obtained with RSEM (RNASeq by expectation maximization) software (http://deweylab.biostat.wisc.edu/rsem/). Differentially expressed genes were identified using edgeR (empirical analysis of digital gene expression data in R) software (http://www.bioconductor.org/packages/release/bioc/html/edgeR.html; Reiner et al., 2003; Robinson and Smyth, 2007). For this analysis, the filtering threshold was set to an FDR (false discovery rate) < 0.5 and a |log2FC| > 1.

All genes were characterized with BALSTX comparisons against the integrated NCBI NR database with an expectation value of 1e-5 (BLAST Version 2.2.28+, http://blast.ncbi.nlm.nih.gov/Blast.cgi). Species information was obtained from the respective taxonomy annotation NR database. The species abundance in each specimen was determined by calculating the FPKM value of each transcript in the respective species. The taxonomic abundance in each specimen was calculated at the domain, kingdom, phylum, class, order, family, and genus levels. Thus, the abundance profiles at the corresponding taxonomic levels were built. Differences in the abundance profile at each level between two groups were identified by Welch's *t*-test using STAMP software (http://kiwi.cs.dal.ca/Software/STAMP).

The meta-transcriptomic sequences were deposited into the NCBI Sequence Read Archive (SRA) under accession numbers SRR4308592, SRR4308642, SRR4330895, and SRR4333242.

### Phylogenetic analysis

A maximum likelihood tree was constructed from the alignment of 12 different 16S rRNA sequences obtained from the GenBank database with MEGA (Molecular Evolutionary Genetics Analysis, version 5) software (Tamura et al., 2007). The names and accession numbers of the related bacterial 16S genes were as follows: *Candidatus Hepatoplasma crinochetorum* (Accession number: AY500249.1), *Mycoplasma pneumonia* (AB680604.1), *Mycoplasma ovipneumoniae* (NR_025989.1), *Ureaplasma parvum* (NR_074762.1), *Ureaplasma cati* (NR_115604.1), *Spiroplasma clarkii* (NR_104750.1), *Spiroplasma eriocheiris* (NR_125517.1), *Mesoplasma florum* (AB681225.1), *Entomoplasma ellychniae* (NR_104951.1), *Entomoplasma luminosum* (AY155670.1), and *Mesoplasma grammopterae* (AY174170.1).

### DNA extraction and polymerase chain reaction

The 16S rRNA gene from *C. Hepatoplasma* was assembled using rRNA raw reads and verified by PCR (polymerase chain reaction) using the primer pair Es.myco.F01 (5′-AGGGTTTGATTATGGCTCAGGA-3′) and Es.myco.R01 (5′-ACAAGACCAGAGAACGTATTCACC-3′). Based on the sequence of the 16S rRNA gene from *C. Hepatoplasma*, a primer pair (Es.myco.F02: 5′-ACTCCTACGGGAGGCAGCAG-3′ and Es.myco.R02: 5′-GCGGCTGCTGGCACATAGTT-3′) was designed to detect this species in the hepatopancreata of crabs infected with HPND. DNA was isolated using the Genomic DNA Extraction Kit according to the manufacturer's protocol (Tiangen, Beijing, China).

## Results

### Reads and assembly statistics

Four meta-transcriptomic libraries were constructed to identify differences in organism communities, especially the bacterial and viral communities from the hepatopancreata of crabs with and without HPND. A total of 195,152,066 reads were generated from four samples, and 162,963 transcripts were assembled (Tables S1, S2). The expression levels of entire transcripts were evaluated (Table S3). The distribution of transcripts to general functional categories was assessed on the basis of best BLAST matches to the COG database, GO database, and KEGG database (Tables S4– S6).

### Taxonomic data and comparison of crabs with and without HPND

Sequences were classified into 60 phyla, which included 16 bacterial phyla. Ten and 11 phyla were found to exist in the hepatopancreata of two crabs with HPND, while 12 and 16 phyla were found to exist in the hepatopancreata of two crabs without HPND. This analysis showed that the diversity of the organism communities in the hepatopancreata of healthy crabs was greater than that in diseased crabs (Table S7). Similar results were found at class, order, family, and genus levels. For example, 572 and 929 genera were identified in crabs with and without HPND, respectively, whereas 544 genera were identified in both groups (Table S8).

**Figure 1:**
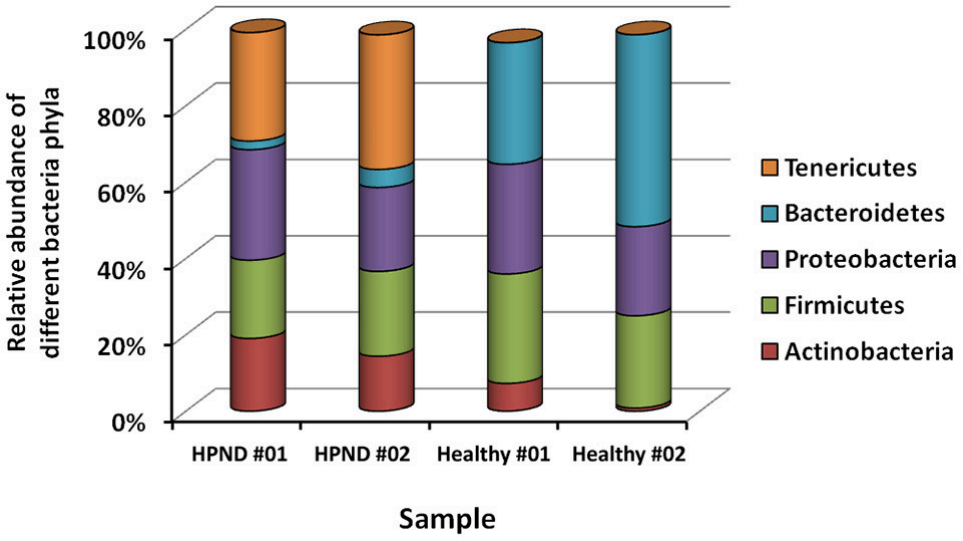
**Relative abundance of bacteria at the phylum level in crabs with and without HPND based on meta-transcriptomic data**. HPND #01, HPND #02: two samples from crabs with HPND; Healthy #01, Healthy #02: two samples from crabs without HPND.

**Figure 2:**
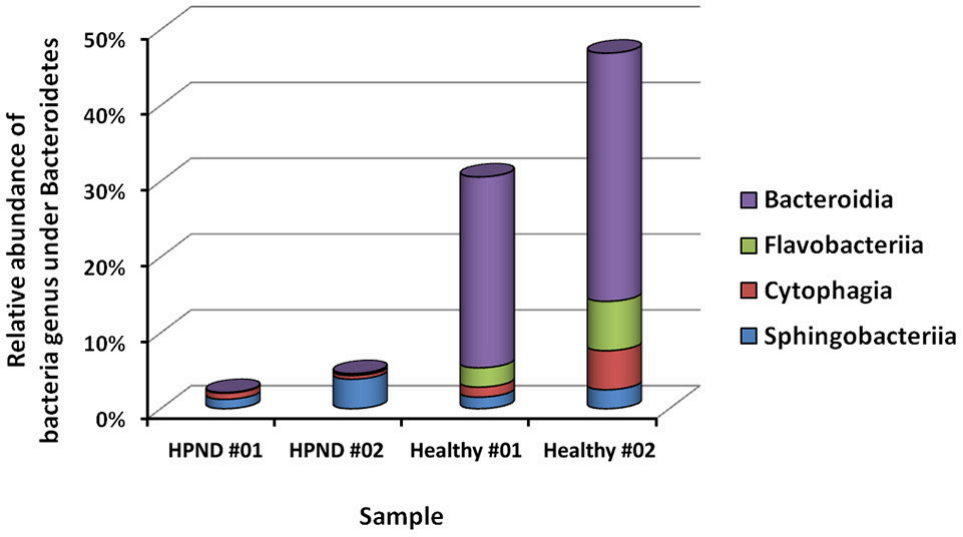
**Relative abundance of bacteria from Bacteroidetes at the class level in crabs with and without HPND**. HPND #01, HPND #02: two samples from crabs with HPND; Healthy #01, Healthy #02: two samples from crabs without HPND.

Among the 16 bacterial phyla, we identified four dominant phyla, namely, Bacteroidetes (41.04%), Firmicutes (26.43%), Proteobacteria (26.03%), and Actinobacteria (4.03%) in the hepatopancreata of crabs without HPND. We also identified five dominant phyla, namely, Tenericutes (31.82%), Firmicutes (21.38%), Proteobacteria (25.45%), Actinobacteria (16.70%), and Bacteroidetes (3.49%) in the hepatopancreata of crabs with HPND. The percentage of bacteria belonging to the Tenericutes phylum was higher in diseased crabs (31.82%) than in healthy crabs (0.02%; *P* < 0.01; Figure 1 and Tables S9, S10). By contrast, the percentage of bacteria belonging to the Bacteroidetes phylum was lower (3.49%) in diseased crabs than in healthy crabs (41.04%). There was a significant decrease in bacteria belonging to the Bacteroidetes phylum in Bacteroidia and Flavobacteria classes in crabs with HPND (Figure 2). In addition, the percentage of bacteria belonging to the Actinobacteria phylum was higher in crabs with HPND (16.70%) than in crabs without the disease (4.03%).

**Figure 3:**
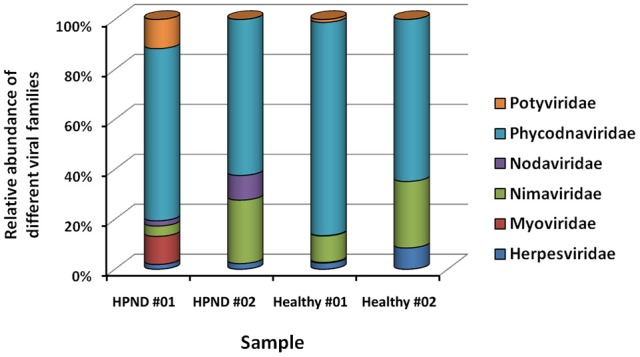
**Relative abundance of viruses at the family level in crabs with and without HPND**. HPND #01, HPND #02: two samples from crabs with HPND; Healthy #01, Healthy #02: two samples from crabs without HPND.

Eleven families of viruses were identified from the analysis of the meta-transcriptome data. The expression levels of these viruses were low in the hepatopancreata of crabs with and without HPND. Five families of virus were present in only one of the crab samples. The remaining six families of virus, which were present in both groups, were compared, and there were no significant differences between groups (Figure 3). We also identified a new nodavirus, whose expression was higher in diseased crabs than in healthy crabs.

**Figure 4:**
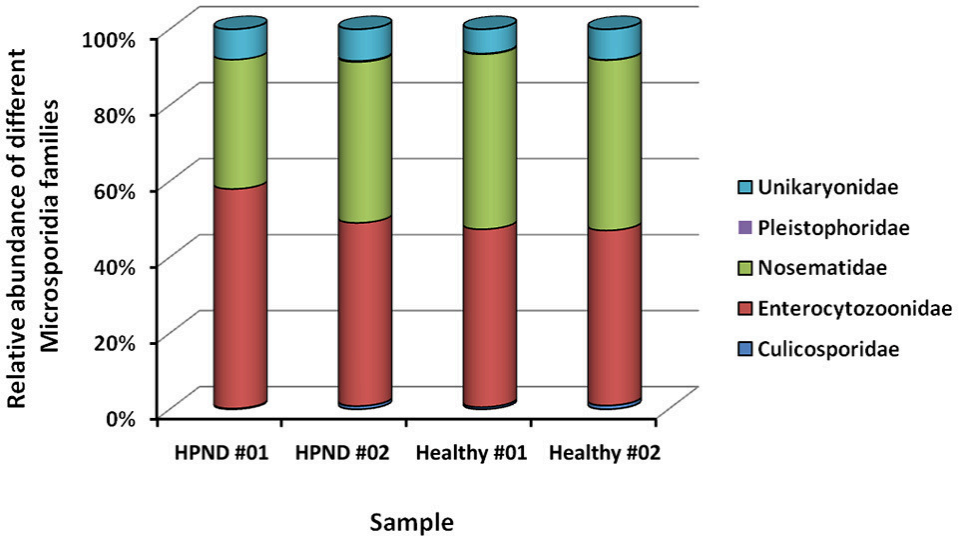
**Relative abundance of Microsporidia at the family level in crabs with and without HPND**. HPND #01, HPND #02: two samples from crabs with HPND; Healthy #01, Healthy #02: two samples from crabs without HPND.

Five families of microsporidia, including three dominant families, were identified from the analysis of the meta-transcriptomic data. These microsporidia were found in the hepatopancreata of crabs with and without HPND, and there were no significant differences between groups (Figure 4).

### Mycoplasmataceae were abundant in crabs with HPND

**Figure 5:**
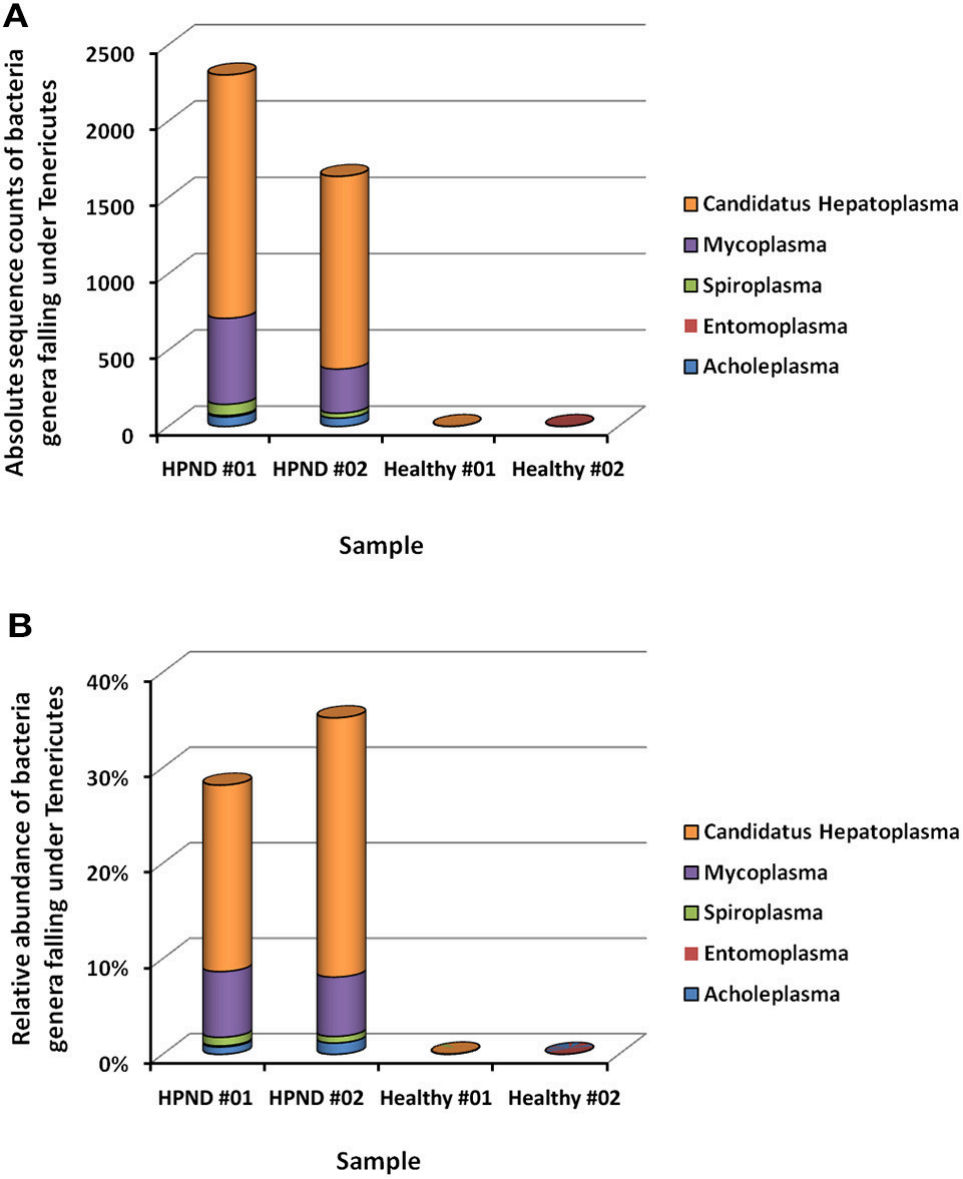
**Bacterial composition from Tenericutes at the genus level in crabs with and without HPND. (A)** Comparison of absolute sequence counts for the major genera of Tenericutes in diseased and healthy crabs. **(B)** Comparison of the relative abundance of the major genera of Tenericutes in iseased and healthy crabs. HPND #01, HPND #02: two samples from crabs with HPND; Healthy #01, Healthy #02: two samples from crabs without HPND.

The different bacteria belonging to the Tenericutes phylum identified in the hepatopancreata of crabs with HPND was further studied by analyzing the meta-transcriptomic data. The majority of the Tenericutes bacteria (>93.8%) belonged to the Mycoplasmataceae family, with 72.9% belonging to the genus *C. Hepatoplasma* and 20.9% belonging to the genus Mycoplasma (Figure 5). In addition, the three genera Acholeplasma (3.05%), Spiroplasma (2.56%), and Entomoplasma (0.19%) were also found in Tenericutes bacterial communities in the hepatopancreata of diseased crabs.

At the gene expression level, 173 protein-coding genes were found to belong to the Mycoplasmataceae family. Compared with healthy crabs, all genes were highly expressed in the hepatopancreata of diseased crabs (Table S11). A BLAST search revealed the majority of these genes (134 genes) to be highly homologous with those from *Candidatus Hepatoplasma crinochetorum*, a species in the genus *C. Hepatoplasma*, family Mycoplasmataceae. The remaining genes (39 genes) were homologous with genes from other species belonging to the Mycoplasmataceae family. For example, an alignment showed all 134 genes to be 33–93% homologous with their corresponding genes in *Candidatus Hepatoplasma crinochetorum*. The gene with the highest homology (93%) was rpoB. These results indicate that these genes are from a microorganism belonging to the genus *C. Hepatoplasma*. For the purpose of this study, this microorganism was designated as “*C*. *Hepatoplasma* sp.”

### Phylogenetic tree analysis

**Figure 6:**
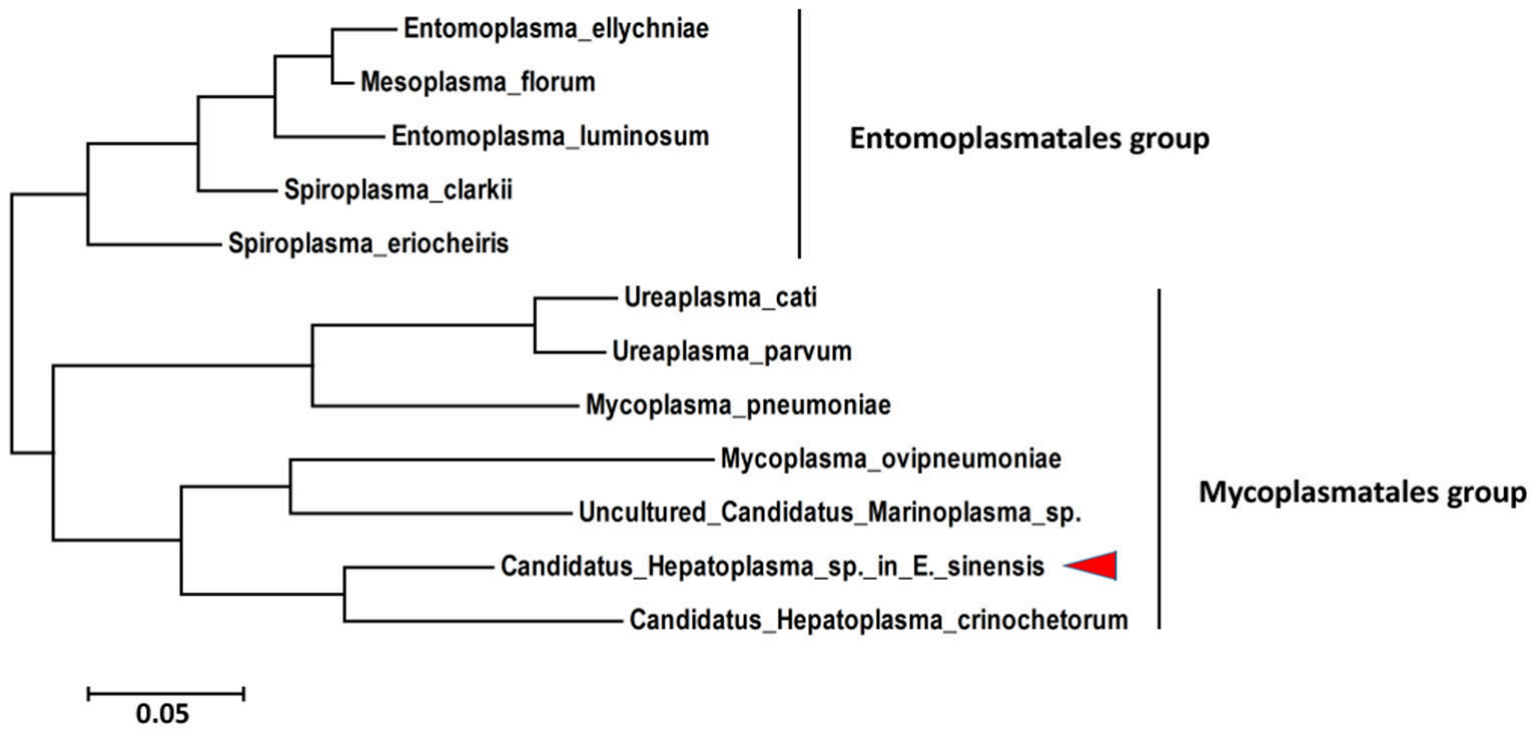
**Phylogenetic tree based on the 16S rRNA gene sequence showing the relationship between ***Candidatus Hepatopancreas*** and Tenericutes**.

The 16S rRNA gene is the most common gene; thus, it was used to identify and to characterize the taxonomic status of the new species described in this study. The partial sequence (1369 bp) of the 16S rRNA gene from *C*. *Hepatoplasma* was cloned from DNA isolated from the hepatopancreata of crabs with HPND. Sequence alignment showed that the 16S rRNA was 87% homologous with that of *Candidatus Hepatoplasma crinochetorum*. Phylogenic analysis indicated that *C. Hepatoplasma* was a close relative of *Candidatus Hepatoplasma crinochetorum* and clustered in one clade (Figure 6). These results confirmed that this microorganism classified under the Mycoplasmataceae family, which was abundant in crabs with HPND, was a new member of the genus *C. Hepatoplasma*.

### Polymerase chain reaction detection

DNA isolated from more than 100 crabs with clinical symptoms typical of HPND was assayed by PCR using a primer pair specific for the 16S RNA gene of *Candidatus Hepatopancreas*. More than 98% of crabs were positive for HPND (Figure S1). Healthy crabs were also screened by PCR, and a weak/no positive band was found.

## Discussion

Meta-transcriptomic analysis provides insights into disease etiology by cataloging and comparing sequences from suspected organisms (Rosales and Thurber, 2015). This approach is powerful, because it simultaneously evaluates both viral and bacterial communities, and even suspected pathogens from the Eukaryota domain. Considering that RNA viruses can associate with certain diseases in *E. sinensis* (Zhang et al., 2004; Zhang and Bonami, 2007, 2012; Shen et al., 2015), and microsporidia were found in crabs infected with HPND (Ding et al., 2016), we analyzed the meta-transcriptomes of hepatopancreata from crabs with and without HPND to identify potential pathogens from viruses, bacteria, and eukaryotes.

We studied the differences between the virome and the microbiome in the hepatopancreata of crabs with and without HPND. Although there was no significant difference in the virome of both groups, we identified a new nodavirus, which was more abundant in the hepatopancreata of diseased crabs than in healthy crabs. The bacterial community in the hepatopancreata of crabs without HPND was more species-rich than that in the hepatopancreata of crabs with HPND. Interestingly, similar findings were reported for the human oral and gut microbiomes during health and disease (Jorth et al., 2014; Ling et al., 2014). We identified four dominant phyla, namely, Proteobacteria, Bacteroidetes, Firmicutes, and Actinobacteria in the hepatopancreata of healthy crabs. A previous study has reported that 90–95% of the bacterial phylotypes in the intestine of *E. sinensis* were Proteobacteria and Bacteroidetes, and that Bacteroidetes were common to all crab types (Li et al., 2007). For example, the phyla Proteobacteria, Bacteroidetes, and Firmicutes were most abundant in the digestive system of the black tiger shrimp *P. monodon* (Shakibazadeh et al., 2009; Chaiyapechara et al., 2012). Similarly, bacteria belonging to the Bacteroidetes phylum were more plentiful in the hepatopancreas, whereas those of the Firmicutes phylum were more plentiful in the foregut and intestine of the shrimp *N. denticulate* (Cheung et al., 2015). In this study, Bacteroidetes, Proteobacteria, and Firmicutes were the most dominant bacterial phyla in the hepatopancreata of healthy crabs, with a combined prevalence of 93%. However, there was a significant difference in the prevalence of the major bacterial phyla in the hepatopancreata of crabs with and without HPND. Tenericutes was the most prevalent bacterial phylum in crabs with HPND crabs (31.82%), whereas its prevalence was low in healthy crabs (0.02%). We also found a significantly low proportion of Bacteroidetes in diseased crabs (3.49%) compared to healthy crabs (41.04%). Interestingly, microbial diversity is lower in obese individuals than in lean individuals, with obese individuals having a lower proportion of Bacteroidetes and a higher proportion of Actinobacteria (Turnbaugh et al., 2009). There was also a negative relationship between the abundance of Bacteroidetes and the presence of fat deposits (Guo et al., 2008). The higher proportion of Bacteroidetes in the hepatopancreas may be related to the higher cellulolytic activity in crabs (Cheung et al., 2015). In this study, a similar shift in the microbial composition was found in the hepatopancreata of crabs with HPND compared with healthy crabs. The evolutionarily-stable commensal microbes positively and negatively affect host health, and when this balance is disrupted, symbiotic microbes can induce disease (Breznak and Brune, 1994; Douglas, 1998; Fraune and Zimmer, 2008; Jorth et al., 2014). The lower proportion of Bacteroidetes and the higher proportion of Actinobacteria indicate that there were abnormalities in the metabolic breakdown of fat and cellulose in the hepatopancreata of crabs with HPND.

The majority of Tenericutes bacteria (>93.8%) belonged to the Mycoplasmataceae family, with 72.9% belonging to *C. Hepatoplasma* and 20.9% belonging to Mycoplasma. Mycoplasmataceae bacteria were found in the symbiotic bacterial community in the gill and the gut of *E. sinensis*. An alignment has shown that *C. Hepatoplasma* shares more than 95% sequence identity with uncultured Mycoplasmataceae detected in the mud crab *Scylla paramamosain* (Li et al., 2012), more than 94% sequence identity with uncultured Mycoplasmataceae bacteria in the carapace, gut, and hemolymph of the Atlantic Blue Crab *Callinectes Sapidus* (Givens et al., 2013), and more than 94% sequence identity with uncultured Mycoplasmataceae symbionts in the midgut of the isopod *Ligia oceanic* (Fraune and Zimmer, 2008). Similar results were reported for the CMC Mollicutes group I that was found in the midgut bacterial community of *E. sinensis* (Chen et al., 2015), suggesting that *C*. *Hepatoplasma* and the CMC Mollicutes group I might belong to the same species of Mycoplasmataceae bacteria. Mycoplasmataceae bacteria were not detected in the guts of crabs from the Yangtze River Estuary and Chongming Islands (Li et al., 2007). Tenericutes were the dominant phyla in the gut of the Chinese mitten crab (Chen et al., 2015). Mycoplasmataceae are one of the most dominant families in the gut; however, they are hardly detected in the gills or water (Zhang et al., 2016). These reports suggest that Mycoplasmataceae bacteria in *E. sinensis* are variable, and that Tenericutes are not indispensable to the crab. A previous study has shown that *Candidatus Hepatoplasma crinochetorum* was beneficial to its isopod host under low-nutrient conditions (Fraune and Zimmer, 2008). In this study, the abundance of *Candidatus Hepatopancreas* in the hepatopancreata of crabs with HPND was indicative of abnormalities in the absorption of nutrients.

We found that the expression of carboxylesterase family genes was significantly up-regulated (Table S12). Carboxylesterase is the major enzyme that breaks down the insecticide pyrethroid, which is widely used in Chinese mitten crab cell cultures, although it is known to harm the hepatopancreas (Wheelock et al., 2006). Further studies are needed to determine how pyrethroid injures the heptopancreas, thus leading to abnormalities in metabolism, nutrient absorption, microbial dysbiosis, and HPND in crabs.

In addition to Mycoplasmataceae, the genera Acholeplasma and Spiroplasma, also from Mullicutes, were abundant in the hepatopancreata of crabs with HPND. Acholeplasma and Spiroplasma bacteria are responsible for crab disease (Wang W. et al., 2004; Chen et al., 2011). An alignment of sequences showed that Spiroplasma and *Spiroplasma eriocheir*, which is believed to cause crab tremor disease, are two different species. Further studies are needed to determine whether Acholeplasma and Spiroplasma associate with HPND infection in crabs.

Microsporidia can also associate with HPND (Ding et al., 2016). Analysis of meta-transcriptome data indicate that the microsporidial community included three major families, namely, Enterocytozoonidae, Nosematidae, and Unikaryonidae. These microsporidia were found in the hepatopancreata of crabs with and without HPND, although there were no significant differences between groups. Regardless, these data indicate that microsporidia are unlikely to cause HPND.

In conclusion, there were statistically significant changes in the microbiota of HPND-infected crabs. Specifically, we found an increased prevalence of bacteria belonging to the Tenericutes and Actinobacteria phyla and a decreased prevalence of bacteria belonging to the Bacteroidetes phylum, thus reflecting abnormalities in metabolism and the absorption of nutrients in HPND-infected crabs and indicating a connection with HPND.

## Author contributions

HS performed the experiment work and statistical analysis, produced the tables and figures, and wrote the paper. YZ, KS, YM, and TD assist the experiment. AS read and made improvements to the manuscript.

## Funding

This study was supported by the Aquatic Three Update Project of Jiangsu Province (Y2016-35) and the Central Public-Interest Scientific Institution Basal Research Fund, Freshwater Fisheries Research Center, CAFS (2017JBFM01).

### Conflict of interest statement

The authors declare that the research was conducted in the absence of any commercial or financial relationships that could be construed as a potential conflict of interest.

## Supplementary material

Figure S1 **PCR detection of the 16S rRNA gene of ***Candidatus Hepatopancreas*** in the hepatopancreata of different crabs**. 01–26: crabs with HPND; ctr01, ctr02: crabs without HPND; M, 500 bp DNA Ladder.

Table S1 **Statistics for sequencing data**.

Table S2 **Statistics of assembly result**.

Table S3 **Gene expression level in different samples**.

Table S4 **The distribution of transcripts to general functional categories was assessed on the basis of best BLAST matches to the COG database**.

Table S5 **The distribution of transcripts to general functional categories was assessed on the basis of best BLAST matches to the GO database**.

Table S6 **The distribution of transcripts to general functional categories was assessed on the basis of best BLAST matches to the KEGG database**.

Table S7 **Abundance of phylum in different samples**.

Table S8 **Abundance of genus in different samples**.

Table S9 *****T***-test between HPND group and Healthy grup in phylum level**.

Table S10 *****T***-test between HPND group and Healthy grup in genus level**.

Table S11 **173 protein-coding genes were annotated belong to the Mycoplasmataceae family**.

Table S12 **Expression level of five carboxylesterase genes in crabs with and without HPND**.
