# Supplementary figures and images for: A Meta-Transcriptomics Survey Reveals Changes in the Microbiota of the Chinese Mitten Crab Eriocheir sinensis Infected with Hepatopancreatic Necrosis Disease
Source: Front Microbiol. 2017 Apr 26;8:732. doi: 10.3389/fmicb.2017.00732 (PMC5405120; doi:10.3389/fmicb.2017.00732)

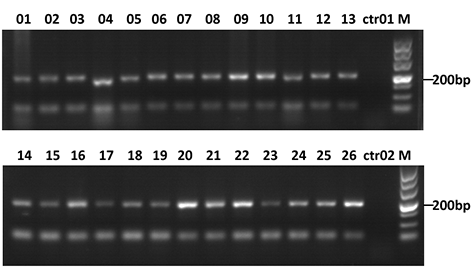

Supplement: Figure S1 — PCR detection of the 16S rRNA gene of Candidatus Hepatopancreas in the hepatopancreata of different crabs. 01–26: crabs with HPND; ctr01, ctr02: crabs without HPND; M, 500 bp DNA Ladder. [file Image1.TIF]
